# A Deep Neural Network for Early Detection and Prediction of Chronic Kidney Disease

**DOI:** 10.3390/diagnostics12010116

**Published:** 2022-01-05

**Authors:** Vijendra Singh, Vijayan K. Asari, Rajkumar Rajasekaran

**Affiliations:** 1 School of Computer Science, University of Petroleum and Energy Studies, Dehradun 248007, India; 2 Electrical and Computer Engineering, University of Dayton, Dayton, OH 45469, USA; vasari1@udayton.edu; 3 School of Computing Science and Engineering, Vellore Institute of Technology, Vellore 632014, India; rrajkumar@vit.ac.in

**Keywords:** chronic kidney disease, feature selection, recursive feature elimination, support vector machine, machine learning

## Abstract

Diabetes and high blood pressure are the primary causes of Chronic Kidney Disease (CKD). Glomerular Filtration Rate (GFR) and kidney damage markers are used by researchers around the world to identify CKD as a condition that leads to reduced renal function over time. A person with CKD has a higher chance of dying young. Doctors face a difficult task in diagnosing the different diseases linked to CKD at an early stage in order to prevent the disease. This research presents a novel deep learning model for the early detection and prediction of CKD. This research objectives to create a deep neural network and compare its performance to that of other contemporary machine learning techniques. In tests, the average of the associated features was used to replace all missing values in the database. After that, the neural network’s optimum parameters were fixed by establishing the parameters and running multiple trials. The foremost important features were selected by Recursive Feature Elimination (RFE). Hemoglobin, Specific Gravity, Serum Creatinine, Red Blood Cell Count, Albumin, Packed Cell Volume, and Hypertension were found as key features in the RFE. Selected features were passed to machine learning models for classification purposes. The proposed Deep neural model outperformed the other four classifiers (Support Vector Machine (SVM), *K*-Nearest Neighbor (*K*NN), Logistic regression, Random Forest, and Naive Bayes classifier) by achieving 100% accuracy. The proposed approach could be a useful tool for nephrologists in detecting CKD.

## 1. Introduction

Chronic kidney disease is a disorder that occurs when a patient’s kidney function deteriorates. As a result, their overall quality of life suffers. Chronic kidney disease affects one out of every 10 people worldwide (CKD). CKD is on the rise, and by 2040, it is expected to be the fifth leading cause of death worldwide [1]. It is one of the leading causes of high medical costs. In high-income nations, the cost of transplantation and dialysis accounts for 2% to 3% of the annual medical budget [2]. Most people with renal failure in low- and middle-income countries have insufficient access to life-saving dialysis and kidney transplants [3]. The number of kidney failure cases is expected to rise unexpectedly in developing countries such as China and India [4]. Chronic kidney failure makes to difficulties in removing extra fluids from the body blood. Advanced chronic kidney disease can cause dangerous levels of fluid, electrolytes, and wastes to build up in the body. It may lead to complications such as high blood pressure, anemia, weak bones, and nerve damage. The strongest indicator of renal function is the Glomerular Filtration Rate (GFR) [5]. Doctors also determine kidney disease through glomerular filtration rate (GFR). The criteria for defining CKD are a kidney damage for ≥3 months with or without decreased GFR or glomerular filtration rate (GFR) less than 60 mL/min/1.73 m^2^ for ≥3 months with or without kidney damage. GFR [6] is the most accurate predictor of kidney function for detecting different phases of CKD, with each phase indicating a more severe reduction in glomerular filtration rate.

The GFR [6] is also used to detect renal failure; if GFR < 15 mL/min, then it means kidney has failed or near to failure. This is the last (fifth) stage of chronic kidney disease. The diagnosis of CKD is a difficult task in medicine because it is based on a variety of symptoms. Clinical judgments are primarily dependent on physicians’ expertise and experience during the examination of the patient’s symptoms [7]. As the health care system evolves and new medicines become accessible, physicians are finding it increasingly challenging to stay up with current clinical practice changes [8].

The machine learning technique provides valid decision-making approaches for computer-assisted automatic disease identification [9]. Machine learning is being used to intelligently interpret available data and transform it into useful knowledge to increase the diagnostic process efficiency [1]. Machine learning is already being used to assess the state of the human body, analyze disease-related aspects, and diagnose a variety of disorders [10]. Heart disease has been diagnosed using models based on machine learning techniques [11]. Diabetes, heart disease, and retinopathy [12], acute renal injury [13], and cancer [14] were all diagnosed using models created by machine learning algorithms. Many researchers have used supervised algorithms, such as Random Forest [15], Fuzzy C Means [16], Naive Bayes [17], Support Vector Machine [18,19,20,21], Gradient Boosting [19,20,21,22], Logistic Regression [20] classifiers in detecting chronic kidney disease. Machine learning can also help to enhance the quality of medical data, reduce the frequency of hospital admissions, and save money on medical expenses. As a result, these models are more commonly utilized in diagnostic analytic research than other older approaches [23]. The only way to reduce chronic disease (CD) mortality is to diagnose it early and treat it effectively [24]. The feature extraction and classification processes in traditional machine learning involve two separate methods. As a result, typical machine learning approaches take a long time to compute. Because of this, the traditional technique is no longer viable for real-time diagnostic applications.

The ability of Artificial Neural Networks (ANN) to learn about fault tolerance, generalization, and the environment is becoming more widespread in the area of medical diagnostics.

In many cases, the Neural Networks (NN) method outperforms standard machine learning techniques. The resource learning architecture can be enhanced to boost its performance even more. The neural networks [25,26,27,28,29] models have been used for the detection of kidney disease. The majority of currently available CKD models have a low classification accuracy. As a result, this research introduces a novel model for Chronic Kidney Disease.

The main contributions of this paper are:

Deep neural networks have been proposed to detect and diagnose CKD.

Apply feature selection to improve efficiency and efficacy of deep neural network.

The computational accuracy of the proposed model is compared with existing classification methods from the literature.

Furthermore, the performance is evaluated through the various performance measures.

The following is a breakdown of the paper structure: The related works on machine learning approaches in the fields of CKD are presented in Section 2. Section 3 presents the proposed deep neural model for early detection of CKD. The results are discussed in Section 4, along with a full explanation. Section 5 wraps up the findings and looks ahead to the future.

## 2. Related Works

Machine learning models have been shown to be effective in predicting and diagnosing serious diseases. Early detection of chronic diseases, particularly the search for new treatments for chronic kidney disease, has gotten a lot of attention from doctors and researchers in recent years. Several recent research has demonstrated that machine learning and deep learning models may be used to successfully diagnose chronic kidney disease (CKD). Table 1 presents a detailed comparison of machine learning methods for the diagnosis of Chronic Diseases from the existing literature.

Z. Chen, X.et al. proved the reliability of multivariate models in clinical practice risk assessment for patients with CKD [30]. The Chronic Renal Failure (CFR) data bank at UC Irvine was used in this investigation. In their comparison investigation, they used the *K*NN, SVM, and Soft independent modeling of class analogy. In comparison to the other two models, the SVM model processed noise within the data set better. In this comparison, the SVM accuracy was 99%. The author of [31] developed a decision-making tool for doctors to forecast the occurrence of CRF in patients. The authors employed *K*NN, Naive Bayes, LDA, random subspace, and tree-based classification techniques on the CRF data set from the UCI repository. The random subspace with the *K*NN classifier has a 94% accuracy rate, according to the researchers. The authors of another study [32] created decision support similar to [31]. The authors classified CRF using Artificial Neural Networks (ANN), Naive Bayes, and decision tree algorithms in this paper. The performance of various machine learning algorithms was examined on Jordan’s Prince Hamza Hospital data set. The decision tree is predicted the most accurate when compared to two other approaches. Song et al. [22] created a gradient boosting-based prediction model to detect CKD using diabetes patient’s EHR and billing data. The authors of [33] published a study on UCI CKD data sets that used SVM, decision trees, Nave Bayes, and *K*NN to detect CKD. The authors developed a ranking algorithm to choose features. With a score of 99.75, the decision tree outperformed three alternative machine learning methods. The authors of [34] presented a hierarchical multiclass classification technique for detecting chronic renal disease in an unbalanced data set.

As a baseline, the authors used naive Bayes, logistic regression, decision trees, and random forests classifiers. Within each patient, the proposed classification approach discovered severe cases. A chronic renal disease diagnosis system was proposed in [35] to diagnose CKD at an early stage. For preparing the data, the authors used the *K*-means technique. On processed data, the *K*NN, SVM, and Naive Bayes classification algorithms were used. Classification algorithms produced the greatest accuracy of 97.8%. Almasoud and Ward [36] reported a study on CKD that used logistic regression, SVM, random forest, and gradient boosting techniques. Four categorization techniques were applied to selected features. Gradient boosting has the highest accuracy of 99%. E M Senan et al. [37] recommended a study on early-stage CKD diagnosis. The RFE method was used to select characteristics from the CKD data set. The outcomes of the SVM, *K*NN, random forest and decision tree algorithms were compared.

Krishnamurthy S. et al. [38] developed various artificial intelligence models to predict Chronic Kidney Disease. The LightGBM model selected the most important features for CKD prediction: age, gout, diabetes mellitus, use of sulfonamides, and angiotensins. The convolutional neural networks achieved the best performance and the highest AUROC metric, 0.954, compared to other models. Mohamed Elhoseny et al. [19] presented an intelligent prediction system for Chronic Kidney Disease. The density-based Feature Selection method eliminates the irrelevant features and then passes selected features to the Ant Colony-based Optimization classifier to predict CKD. Singh and Jain [39] presented novel hybrid approach for diagnose CKD and achieved 92.5 % of prediction accuracy. An artificial neural network for CKD diagnosis was proposed by Neves et al. [25]. The diagnostic sensitivity values ranged from 93.1% to 94.9%, and the diagnostic specificity values ranged from 91.9% to 94.2% in this work.

Vasquez-Morales et al. [27] used large CKD data to generate a neural network classifier, and the model was 95% accurate. Makino et al. [28] used textual data to extract patients diagnoses and treatment information in order to forecast the course of diabetic kidney disease. Ren et al. [29] developed a predictive model for the identification of CKD from an Electronic Health Records (EHR) data set. This proposed model is based on a neural network framework that encodes and decodes the textual and numerical information from EHR. Ma F. et al. [40] develop a deep neural network model to detect chronic renal disease. The presented model obtained the highest accuracy compared to ANN and SVM. Almansour et al. [41] devised a way for preventing CKD using machine learning. The SVM and ANN were among the machine learning classification algorithms used by the researchers. The results of the experiments revealed that ANN had a greater accuracy of 99.75% than SVM.

J. Qin et al. [42] presented a machine learning method for the early detection of CKD. They used logistic regression, random forest, SVM, naive Bayes classifier, *K*NN, and feed-forward neural network to develop their models. The most accurate classification model was random forest, which had a 99.75% accuracy rate. Z. Segal et al. [43] presented a machine learning technique based on an ensemble tree (XGBoost) for the early diagnosis of renal illness. The presented model was compared against Random Forest, CatBoost, Regression with Regularization. The proposed model showed better performance in all matrices, including c-statistics 0.93, sensitivity 0.715, and specificity 0.958. Khamparia et al. [44] developed a deep learning model for early detection of CKD, in which features were selected from multimedia data using a stacked autoencoder model. The authors used A SoftMax classifier to predict the final class. It was observed that the proposed model achieved the highest performance in comparison to conventional classification techniques on the UCI CKD data set.

Polat, H. et al. [45] presented a study on the role of effective feature selection methods in the accurate prediction of CKD. In this paper, wrapper and filter feature selection approaches were used to select the dimension of the Chronic Kidney Disease data set. The selected features are then passed to Support Vector Machine to classify Chronic Kidney Disease for diagnosis purposes. The experimental results presented that Support Vector Machine generates better results on selected features by the Best First search method with filtered subset evaluator. SVM achieved an accuracy rate (98.5%) in comparison to features selected by other wrapper and filter methods. Ebiaredoh-Mienye Sarah A. et al. [46] presented a robust model for the prediction of CKD that integrates an enhanced sparse autoencoder (SAE) and Softmax regression. In this proposed model, autoencoders achieved sparsity by penalizing the weights. The Softmax regression was optimized for the classification task; therefore, the proposed model achieved excellent performance. The proposed model obtained an accuracy of 98% on the chronic kidney disease (CKD) data set. The proposed model achieved comparable performance with other existing methods. Zhiyong Pang et al. [47] proposed a fully automated computer-aided diagnosis system to classify malignant and benign masses using breast magnetic resonance imaging. The texture features were selected by integration of support vector machine with ReliefF feature selection method. This system achieved an accuracy of 92.3%. Chen et al. [21] presented a model in which Hepatitis was diagnosed with a hybrid method that integrates a Fisher discriminatory analysis algorithm and an SVM classifier. As a result of comparing the proposed method with the existing methods, the hybrid method outperforms the other methods, and the highest classification accuracy of 96.77% is achieved. The authors presented a breast cancer diagnosis model in this study [48]. The selected features by sequential forward selection and the backward selection methods are passed to Artificial Neural Networks to classify breast cancer. SBSP + NN achieved the highest accuracy of 98.75%.

**Table 1:** Comparative Accuracy analysis of the diagnosis of Chronic Diseases from literature.

Chronic Diseases Diagnosis	Model	Accuracy Achieved (%)	Reference
Kidney Renal Failure	Artificial Neural Networks	91.9%–94.2%	[25]
Diabetic Kidney Disease	Convolutional Model	71%	[28]
Chronic Kidney Disease	Neural Network Classifier	95%	[27]
Breast Cancer	SBSP + NN	98.57%	[48]
Hepatitis Disease	FDA and SVM	96.77%	[21]
Breast Cancer	SVM + ReliefF	92.3%	[47]
Chronic Kidney Disease	*K*NN, SVM	99%	[30]
Chronic Renal Failure	Fisher Discriminatory Analysis and SVM	96.7%	[21]
Chronic Renal Failure	*K*NN, Naive Bayes, LDA, Random Subspace and Tree-Based Decision	94%	[31]
Chronic Kidney Disease	SVM, *K*NN, and Naïve Bayes Decision tree,	99.7%	[33]
Chronic Kidney Disease	Logistic Regression, Decision Tree, Naïve Bayes, and Random Forests	93%	[34]
Chronic Kidney Disease	*K*NN, SVM, and Naïve Bayes	97.8%	[35]
Chronic Kidney Disease	SVM, *K*NN, and decision tree	99.1%	[37]
Chronic Kidney Disease	Convolutional Neural Networks	95.7%	[38]
Chronic Kidney Disease	SVM, Random Forest, and Gradient Boosting	99%	[36]
Chronic Kidney Disease	Logistic regression, *K*NN, SVM, Random Forest, Naive Bayes and ANN	99.7%	[42]
Chronic Kidney Disease	XGBoost	95.8%	[43]
Chronic Kidney Disease	SVM	98.5%	[45]
Chronic Kidney Disease	Softmax Regression	98%	[46]

## 3. Materials and Methods

### 3.1. Data Set Description

The University of California Irvine (UCI) Repository was used to gather CKD data. There are 400 patient records in the data set, and some values are missing. It comprises 24 clinical qualities that emerge in the prognosis of chronic kidney disease, with one class attribute serving as a result of the patient’s presence of chronic renal failure being predicted. There are two types of values in the expected feature diagnostic: “ckd” and “notckd.” The data set contains 250 values of the “ckd” class (62.5%) and 150 values of the “notckd” class (37.5%). The characteristics of the UCI CKD data collection are listed in Table 2.

### 3.2. Data Processing

The estimation of missing values and the removal of noise such as outliers, as well as the normalization and validation of unbalanced data, were all part of the preprocessing stages. When assessing a patient, some measurements could be missing or incomplete.

#### 3.2.1. Handling Missing Values

There are 158 completed cases in the data collection, with the remainder missing. Ignoring records is the simplest technique to deal with missing values; however, this is not practical for small data sets. The data set is examined during the data preparation process to see whether any attribute values are missing. The missing values for numerical features were estimated using the statistical technique of mean imputation. The mode technique was used to replace the missing values of nominal features.

#### 3.2.2. Categorical Data Encoding

Because most machine learning algorithms only accept numeric values as input, category values must be encoded into numerical values. The binary values “0” and “1” are used to represent the characteristics of categories such as “no” and “yes”.

#### 3.2.3. Data Transformation

Data transformation is the process of transforming numbers on the same scale so that one variable does not dominate the others. Otherwise, learning algorithms perceive larger values as higher and smaller values as lower, regardless of the unit of weight. Data transformations alter the values in a data set so that they can be processed further [49]. To improve the accuracy of machine learning models, this research employs a data normalization technique. It converts data between the −1 and +1 ranges. The converted data has a standard deviation of 1 and a mean of 0.

The standardization formula is given below:

(1) $$w=\frac{\left(x-\bar{x}\right)}{\sigma }$$

where,

*w* = Standardized score

*x* = Observed value

$\bar{x}$ = Mean

*σ* = Standard deviation

#### 3.2.4. Outlier Detection

Outliers are observation points that are isolated from the rest of the data. An outlier could be caused by measurement variability or signal an error in the experiment. An outlier can distort and mislead the learning process of the machine learning algorithm. It leads to longer training times, less accuracy in the model, and ultimately to poorer results. This paper uses the Interquartile Range (IQR) [49] based approach to remove outliers before transferring data to the learning algorithm.

### 3.3. Feature Selection

Recursive Feature Elimination (RFE) removes features recursively, building a model based on other features [50]. It applies greedy search to find the most efficient subset of features. Use model accuracy to determine which features are most appropriate for predicting a feature. It develops models iteratively, determining the best or worst feature for each iteration. The traits are then classified based on the sequence in which they were removed. If the data set contains N functions, recursive feature elimination will eagerly search for a combination of 2N features in the worst-case scenario.

### 3.4. Classifiers

#### 3.4.1. Support Vector Machine

**Figure 1:**
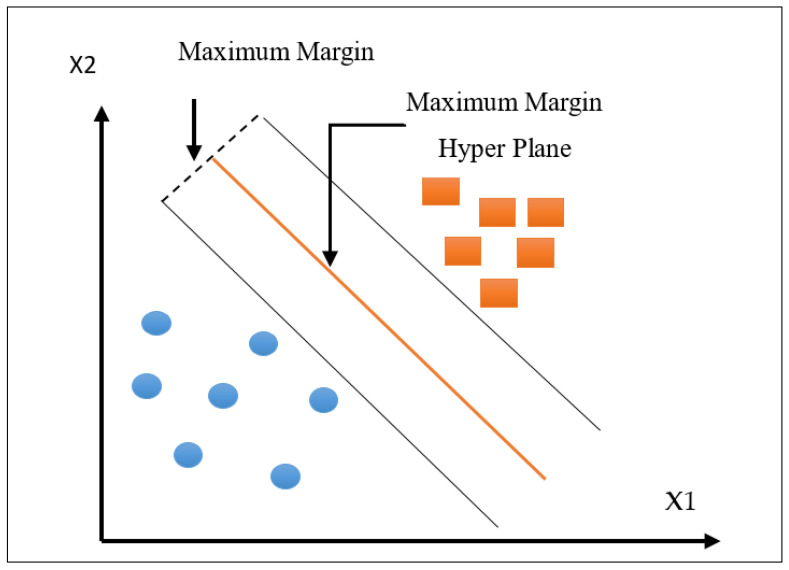
Support Vector Machine.

The SVM constructs a separation hyperplane that splits the labeled data into classes and determines whether a new data value belongs above or below the line. There may be several hyperplanes, and the one with the largest margin between data points is chosen. Figure 1 shows the maximum hyperplanes and maximum margin of the support vector machine. The equation of hyperplane that separates two classes is given by:

(2) $$D\left(x\right)\;=w_{0}+w_{1}a_{1}+w_{2}a_{2}$$

However, the equation of the maximum-margin hyperplane can be written

(3) $$x=b+\sum_{i}\propto_{i}y_{i}a\left(i\right)\;\times \;a$$

Here, *i* is the support vector, and *y_i_* is the training instance *a*(*i*) class value. The learning algorithm determines the numeric value *b* and *α_i,_* respectively.

#### 3.4.2. *K*-Nearest Neighbor

**Figure 2:**
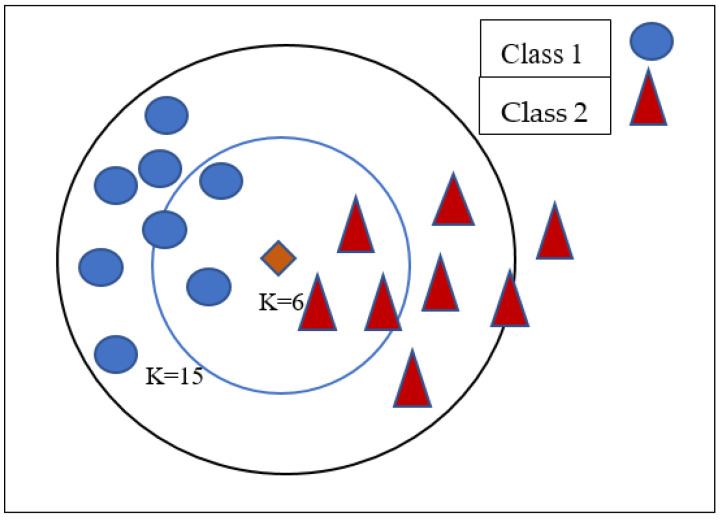
*K*-Nearest Neighbor.

The *K*NN algorithm recognizes similarities between new and previous data points and categorizes fresh test points into existing related groups. The *K*NN method is a slow learning algorithm since it is not parametric. This means that instead of learning from the training data set, it should be secured. It uses *K* to categorize the data. The distance between the new location and the saved training point was determined using the Euclidean distance. Figure 2 depicts *K*-Nearest neighbor classification based on *K* values.

(4) $$d_{ij}=\sum_{t=1}^{n}{\left(x_{it}^{test}-x_{jt}^{train}\right)}^{2}$$

*K*NN algorithm searches *t* training data set with minimum distance to the testing set.

#### 3.4.3. Decision Tree Classifier

**Figure 3:**
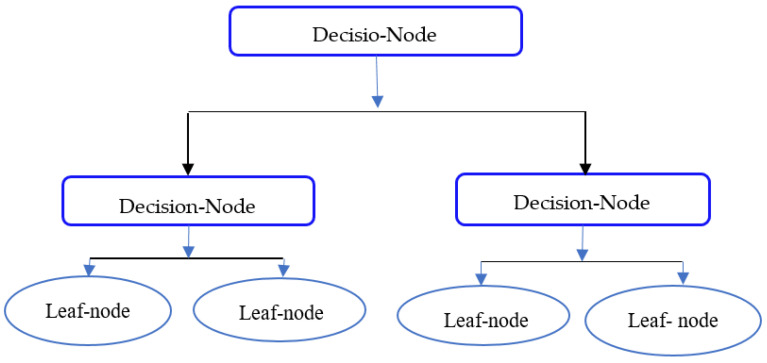
Decision trees.

Decision trees are a nonparametric method of supervised learning [51]. This is a classified structured tree that defines the characteristics of a data set. It represents internal rules for decision-making through internal nodes and tree branches. It has two types of nodes, the decision, and the leaf nodes. The decision nodes take some decisions, and the outcomes of such decisions are leaf nodes. A decision tree has presented in Figure 3.

#### 3.4.4. Random Forest Classifier

**Figure 4:**
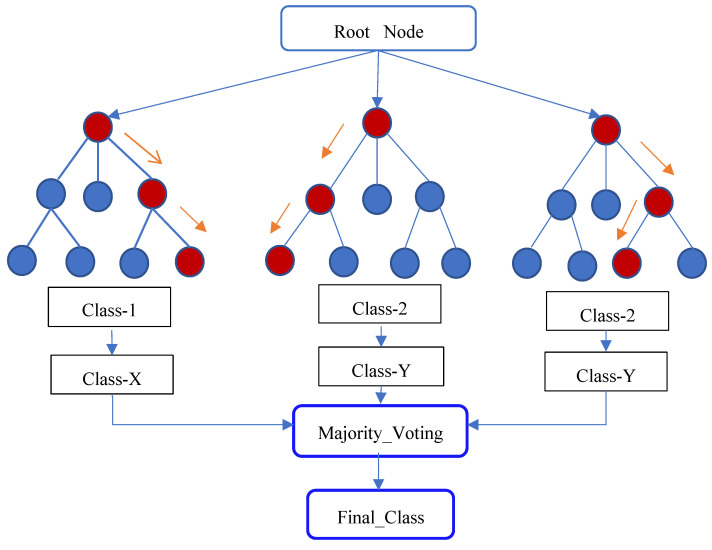
Random Forest.

The random forest algorithm is based on ensemble learning, improving the model’s performance, and solving complex problems by combining several classifiers. A classifier named after the algorithm that contains multiple decision trees averaged over a database subset to improve predictions. In the forecasting process, it does not rely on a single decision tree, and the random forest algorithm creates forecasts from each decision tree that predicts the conclusion based on the majority of decision votes. The usage of several trees decreases the possibility of the model overfitting. To predict the classes in the database, the algorithm includes many decision trees, some of which can predict the proper outcome while others cannot. As a result, there are two assumptions regarding the prediction’s accuracy. To forecast a more accurate outcome than an estimate, the algorithm must first include the actual value of the feature variable. Second, there must be an extremely low correlation between the forecasts for each tree. As a result, there are two requirements for high forecast accuracy. Figure 4 shows a Random Forest Classifier.

### 3.5. Model Development

**Figure 5:**
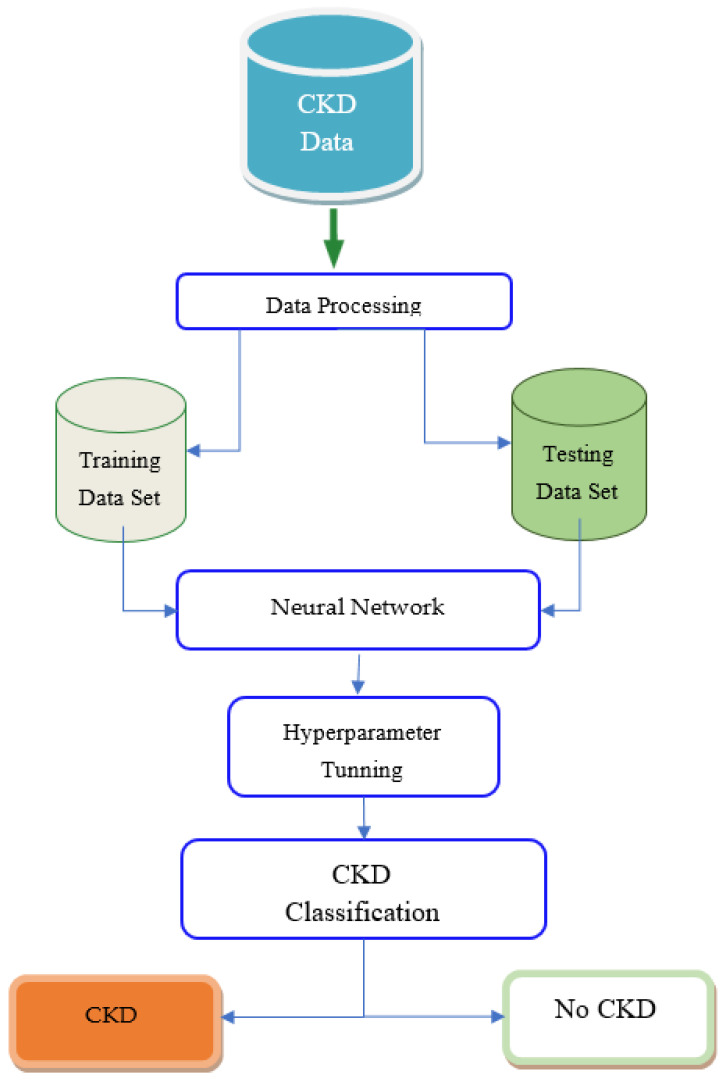
A framework of the proposed model.

Figure 5 depicts the model’s framework. Preprocessing, model hyper tuning, and classification are the three phases of the proposed model. Because the data set may contain noise and redundant values, the preparation step is the most important.

**Figure 6:**
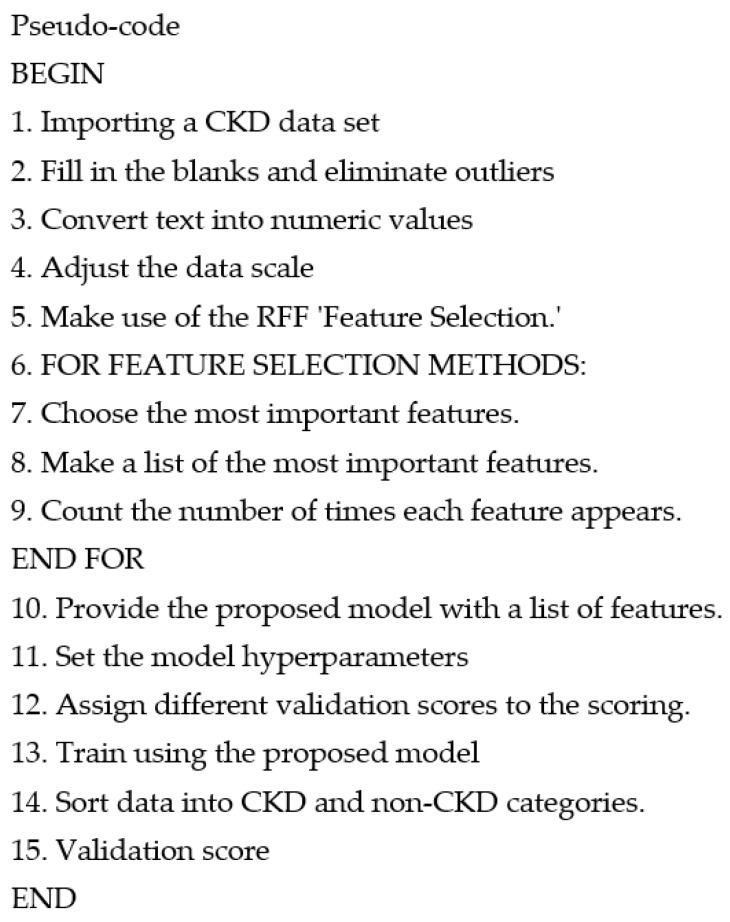
Pseudo-Code of the proposed model.

This phase applied different methods such as handling missing values, categorical data encoding, data transformation, removing outliers and extreme values, and feature selection. The CKD data set is separated into training and testing data sets after being preprocessed. Only a few features are selected using Recursive Feature Elimination out of a total of 24 features in this study. The RFE algorithm evaluates each feature’s value based on its significance, which helps to lower the method’s processing complexity. Finally, redundant and unrelated characteristics are filtered away. The learning model is then fed with the most important characteristics. Figure 6 shows the pseudo-code for the proposed methodology. Initially, a method was introduced to prepare and standardize the data in the data set. The processed data is further passed for processing.

**Figure 7:**
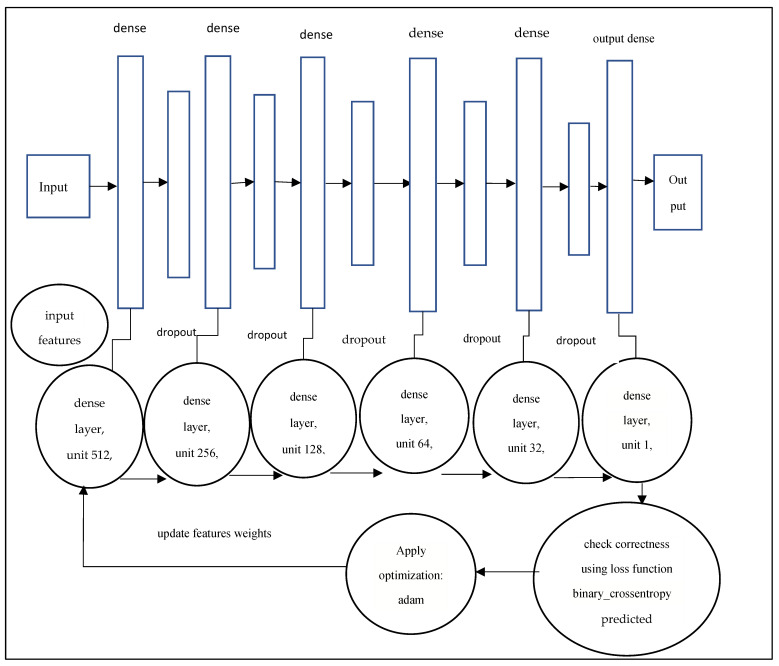
Layers architecture of the proposed model deep neural network.

There are 12 layers in the proposed model architecture: an input layer, five dense layers, five drop layers, and an output dense classifier layer. In Figure 7, the layered architecture’s exact specifications are depicted. Each dense layer is connected directly in a feed-forward method in this architecture. The layer is built in such a way that the outputs of its activation maps are handed on to all following levels as input. A dropout layer is placed between two dense layers in this model, with drop rates of 0.5, 0.4, 0.3, 0.2, and 0.1. Figure 7 presents the layered architecture of the proposed model.

The CNN model has several hyperparameters that need to be optimized. The optimal hyperparameters selection process is experimental; however, it is time-consuming and difficult. Adam [52,53] optimizer initiates hyperparameters with smaller parameters during the training phase.

Adam uses adaptive assessment to determine individual learning rates for various hyperparameter grades ranging from first to second-order gradients. Stochastic Gradient Optimization (SGD) [54] is less efficient than Adam. It necessitates minimal learning time and memory. The classification performance is enhanced by the CNN correct activation function. Neural network’s standard activation functions are sigmoid, tan, Rectified Linear Unit (ReLU) [55], Exponential Linear Unit (ELU) [56], and Self-Normalized Linear Unit (SELU) [57]. This paper tested the different activation functions on the CKD data set and selected the preferred one in all the models.

## 4. Results and Discussion

### 4.1. Experiment Setup

The proposed model was created using data from a variety of situations. The configuration of the system of the developing model is shown in Table 3.

### 4.2. Evaluation Parameters

The proposed model accuracy was calculated by making the CKD class value positive and the notCKD class value negative. The confusion matrix was utilized to evaluate the performance by using True Positives (TP), True Negatives (TN), False Positives (FP), and False Negatives (FN) [58]. According to TP, CKD samples have been accurately categorized. The findings of the FN test show that CKD samples were misclassified. The notCKD samples were not accurately identified, as indicated by a false-positive result (FP). True negative (TN) samples have been accurately categorized as not CKD.

#### 4.2.1. Accuracy

It refers to the proportion of correct guesses to total predictions. Accuracy can be described as the ability to accurately predict the outcome of a situation.

(5) $$\mathrm{Accuracy}=\frac{T_{P}+T_{N}\;}{T_{P}+T_{N}+F_{P}+F_{N}\;}$$

#### 4.2.2. Recall

The recall calculates the proportion of accurately predicted positive observations to the total number of observations in the class, as shown in the following equation.

(6) $$\mathrm{Recall}=\frac{T_{P}}{T_{P}+F_{N}}$$

#### 4.2.3. Specificity

The specificity estimates the number of well-scored negative patterns. The higher the specificity value, the more negative the classifier. It can be defined as:

(7) $$\mathrm{Specificity}=\frac{T_{N}}{T_{N}+F_{P}}$$

#### 4.2.4. Precision

As stated in the equation below, this metric represents the proportion of accurately predicted positive observations to total predictive positive observations.

(8) $$\mathrm{Precision}=\frac{T_{P}}{T_{P}+F_{P}\;}$$

#### 4.2.5. F-Measure

Precision and Recall are weighted averaged in the F-measure [58]. False positives and false negatives are part of the process. F-measure is a term that is defined as

(9) $$\text{F-Measure}=\frac{\mathrm{Two}\times \;\left(\mathrm{Precision}\times \mathrm{Recall}\right)}{\left(\mathrm{Precision}+\mathrm{Recall}\right)}$$

The F-Measure values lie from 0 to 1.

### 4.3. Comparative Analysis of Results

**Figure 8:**
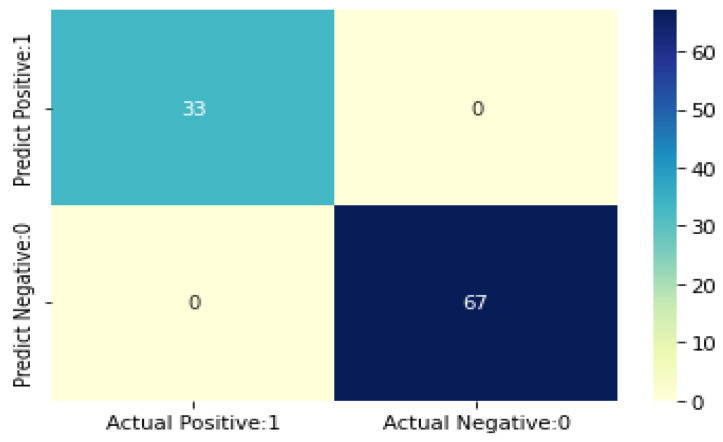
Confusion matrices of the Proposed model.

The findings of the proposed model are presented in this section. The CKD data sets are split into 75% training and 25% test data sets. The hyperparameter settings for the proposed model are shown in Table 4. The confusion matrices are shown in Figure 8. It demonstrates that the suggested model correctly identified all genuine positive and true negative events. The CKD class reports recall, precision, sensitivity, F1 score, and accuracy.

**Figure 9:**
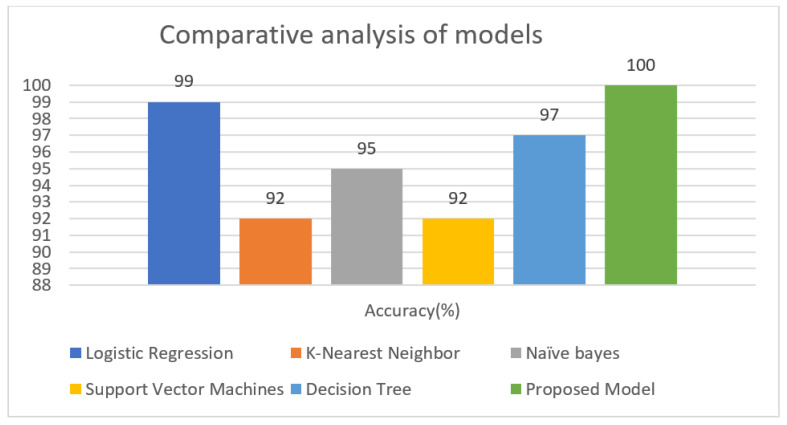
Accuracy graphical representation for the UCI CKD data set.

**Figure 10:**
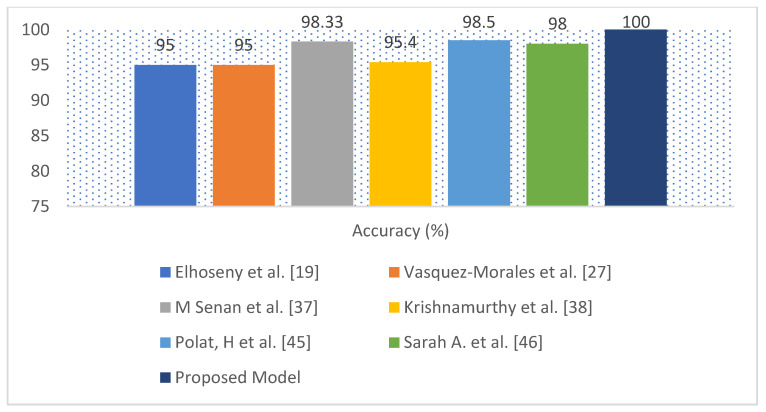
Accuracy graphical representation for the UCI CKD data set.

The proposed model is compared with other classifier algorithms, including logistic regression, *K*NN, SVM, Decision tree, and Random forest. No parameter adjustments were made for these algorithms to show the improved performance of the proposed model. Therefore, the default value for a parameter was used in scikit-learn. All models are evaluated using the F1-score. Table 5 and Table 6 showed experimental results when the proposed model was tested on CKD data sets. In contrast,

Figure 9 and Figure 10 depict accuracy graphs comparing the performance of existing classification algorithms to the proposed approach for chronic kidney disease prediction.

The accuracy of *K*NN, SVM, Naïve Bayes, Decision tree, logistic regression, and the proposed model is 92%, 92%, 95%, 97%, 99% and 100%, respectively. The proposed model was found to be the most accurate, with a 100% accuracy rate. Because it optimally identified positive samples as 250 samples (TP) and all 150 samples as negative samples, the suggested model appropriately classifies all positive and negative samples (TN). True Positive samples were graded 99%, 92%, 95%, 92%, and 97% by Logistic Regression, *K*NN, Nave Bayes, SVM, and Decision Tree, respectively, with a margin of error of 1%, 8%, 5%, 8%, and 3%, respectively. The results of all five classifiers are shown in Table 5.

The proposed model outperforms the other classifiers by scoring 100% on all measures. The F1-score, accuracy, precision, and recall of the Logistic regression were all 99%, 99%, 100%, and 98%, respectively. Then Decision Tree obtained an F1-score, Accuracy, Precision, and Recall of 97%, 97%, 95%, and 100%, respectively. The Naïve Bayes F1-score, Accuracy, Precision, and Recall values were 95%, 95%, 92%, and 100%, respectively. The F1-score, Accuracy, Precision, and Recall values of Naïve Bayes were 92%, 92%, 88%, and 98%, respectively. The Support Vector Machines classifier performed the lowest with F1-score, Accuracy, Precision, and Recall values of 92%, 92%, 87%, and 96%, respectively.

Table 6 compares the proposed model to several recent scholarly studies, such as Ant Colony-based Optimization Classifier by Elhoseny et al. [19], Neural network by Vasquez-Morales et al. [27], *K*NN by M Senan et al. [37], Convolutional Neural Networks by Krishnamurthy et al. [38], SVM by Polat, H. et al. [45], and SAE and Softmax Regression proposed by Sarah, A. et al. [46]. The proposed model has obtained an accuracy of 100%, while the exiting works obtained the accuracy from 85% to 98.5%. Finally, it should be noted that the proposed model is more efficient than existing classification methods.

### 4.4. Feature Importance from RFE

**Figure 11:**
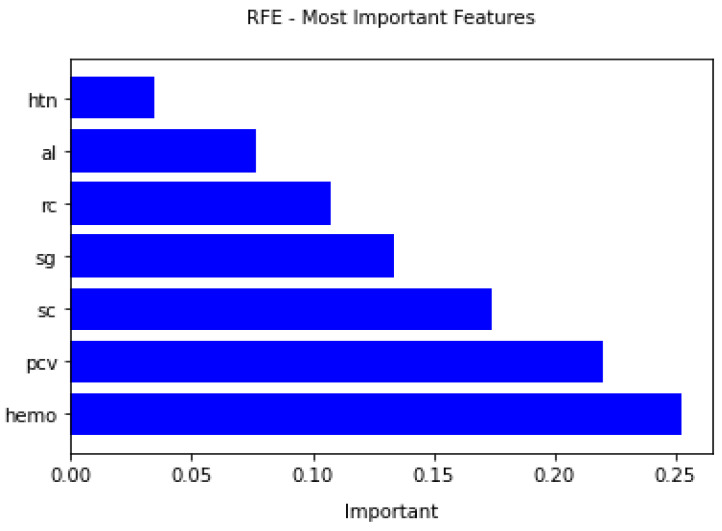
Important features selected by RFE.

This section of the paper presents the most important feature selected by the RFE algorithm based on their ranking. The figure shows the chosen features and their importance during the classification of CKD disease. The most critical risk factors are Hemoglobin, Serum Creatinine, Specific Gravity, Packed Cell Volume, Red Blood Cell Count, Hypertension, and Albumin, as presented in Table 7. The nephrologists should focus on these risk factors while diagnosing CKD disease patients. Figure 11 shows feature selected by RFE with their importance.

### 4.5. Receiver Operating Characteristic (ROC)/Area under Curve (AUC)

**Figure 12:**
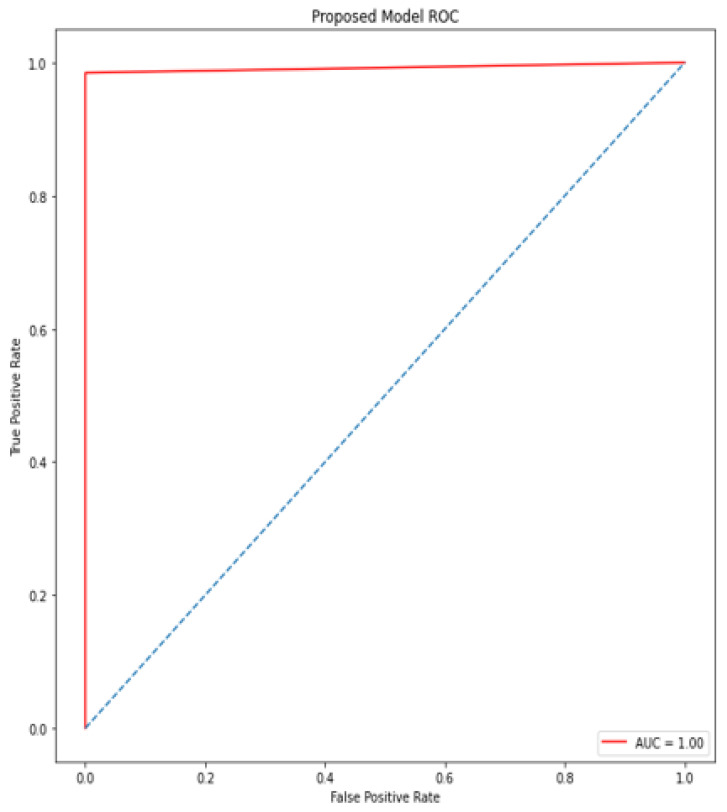
ROC/AUC of Proposed model.

**Figure 13:**
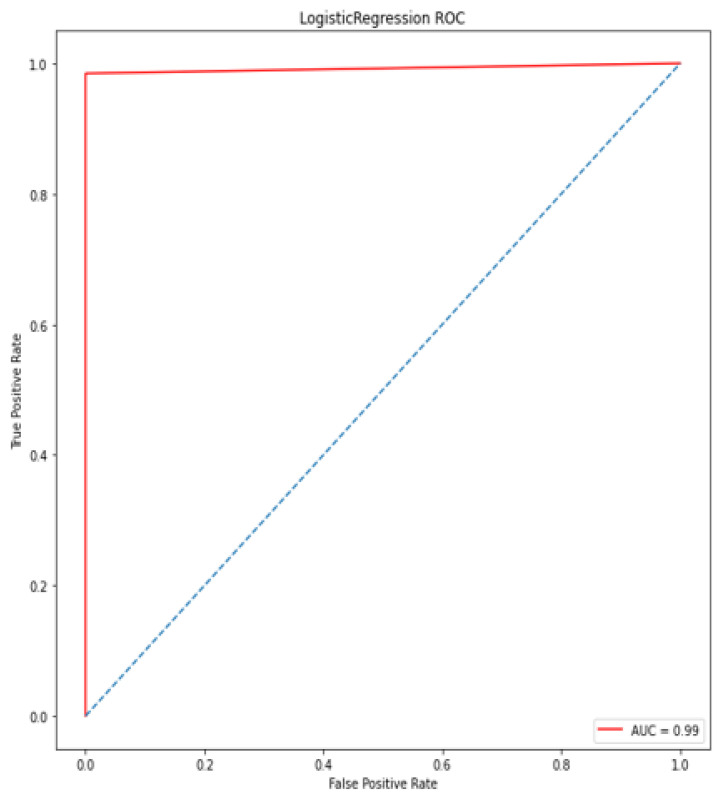
ROC/AUC of Logistic Regression.

**Figure 14:**
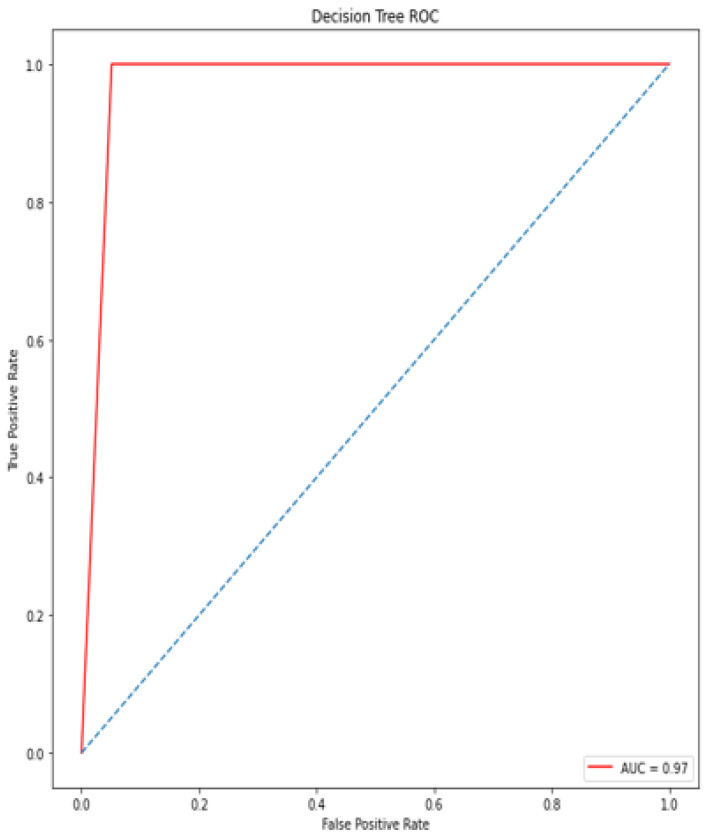
ROC/AUC of Decision tree.

**Figure 15:**
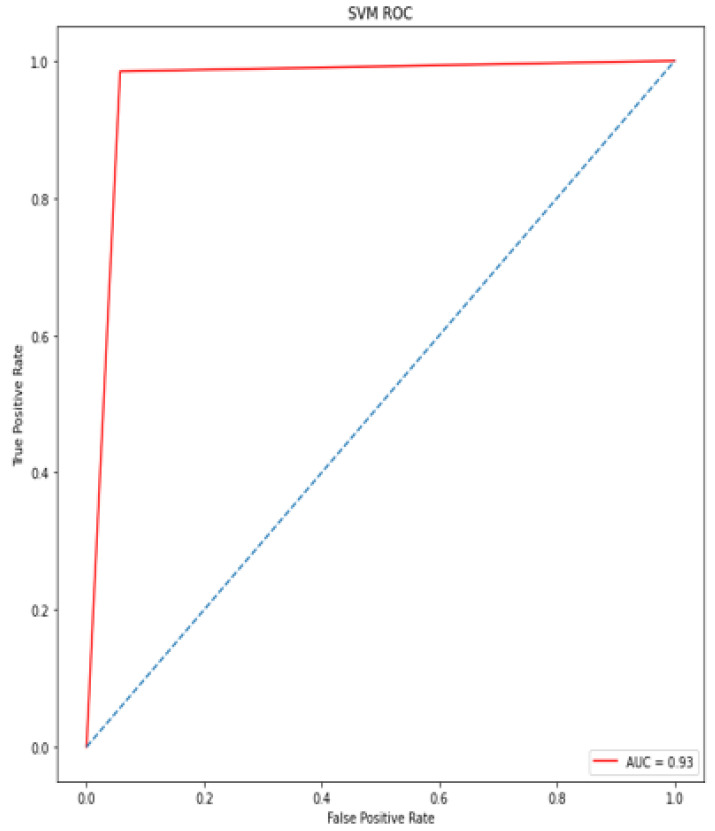
ROC/AUC of SVM.

**Figure 16:**
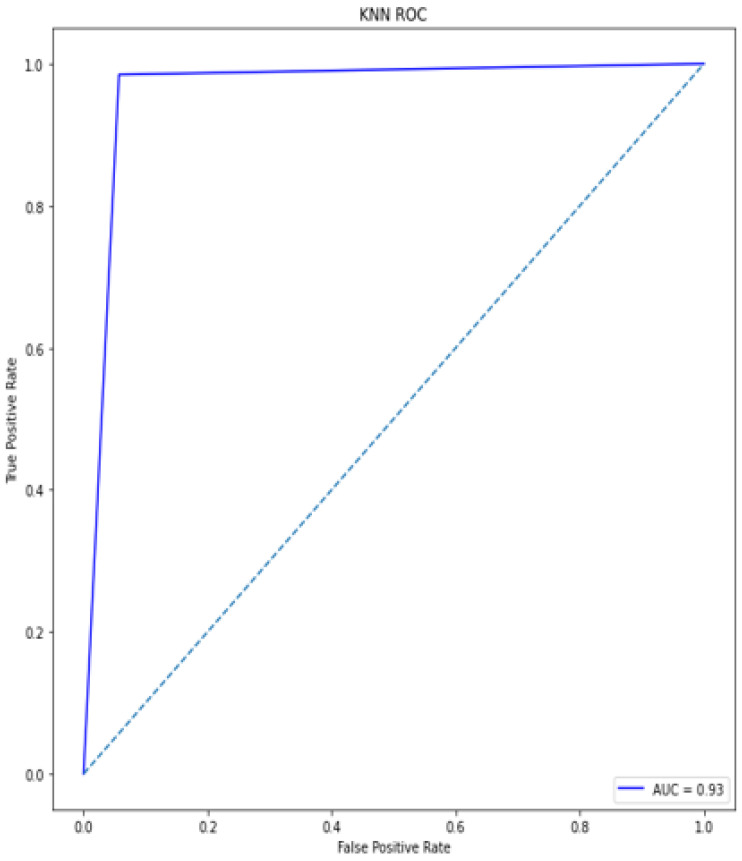
ROC/AUC of *K*NN.

**Figure 17:**
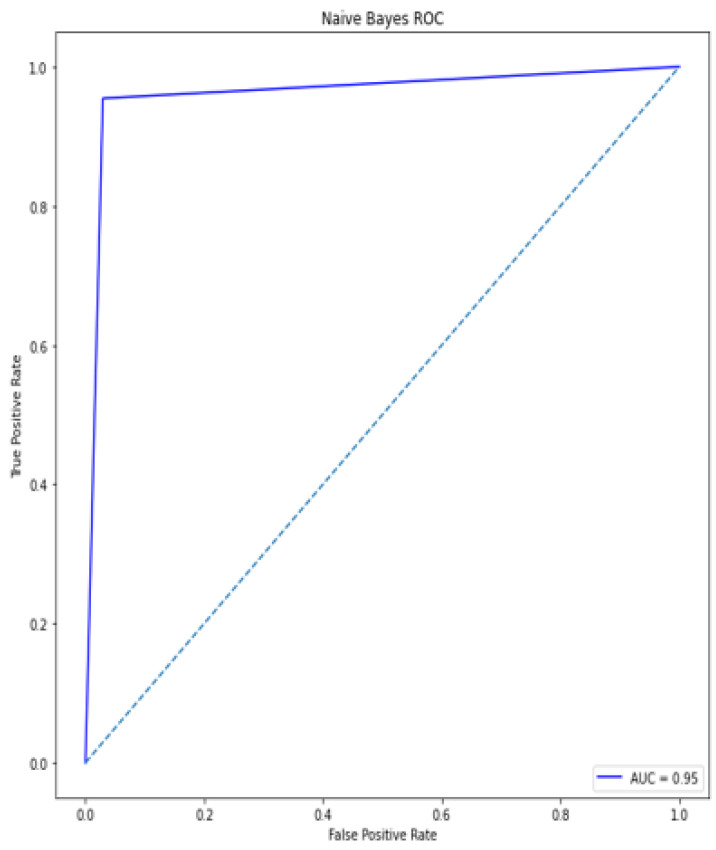
ROC/AUC of Naïve bayes.

The bottom of the square and the ROC curve define the area of the AUC. AUC scores closer to 1 indicate good performance, whereas AUC scores closer to 0.50 indicate poor performance. Figure 12, Figure 13, Figure 14, Figure 15, Figure 16 and Figure 17 shows the ROC/AUC curve of the proposed model, logistic regression, Decision tree, SVM, *K*NN, and Naïve Bayes respectively. The proposed model achieved the highest AUC score value 1.0.

## 5. Conclusions and Future Work

A deep learning model for the early diagnosis of chronic disease is presented in this work. In this research, the authors looked at the Recursive Feature Elimination approach to identify which features are the most important for prediction. The most essential CKD features are packed red blood cell count, albumin, cell volume, serum creatinine, specific gravity, hemoglobin, and hypertension. Classification algorithms are fed with a set of features. Different metrics, including classification accuracy, recall, precision, and f-measure, are used to estimate the comparative analysis. The proposed deep neural model outperformed the other five classifiers (Support Vector Machine (SVM), *K*-Nearest Neighbor (*K*NN), Logistic regression, Random Forest, and Naive Bayes classifier) by achieving 100% accuracy. The accuracy of *K*NN, SVM, Naïve Bayes, Decision tree, Random Forest, logistic regression is 92%, 92%, 95%, 97%, and 99%, respectively.

The performance of the proposed model compared with several recent scholarly studies, such as Ant Colony-based Optimization Classifier by Elhoseny et al. [19], Neural network by Vasquez-Morales et al. [27], *K*NN by M Senan et al. [37], Convolutional Neural Networks by Krishnamurthy et al. [38], SVM by Polat, H. et al. [45], and SAE and Softmax Regression proposed by Sarah A. et al. [46]. The exiting works obtained the accuracy from 85% to 98.5%, while the proposed model has obtained an accuracy of 100%. The proposed approach could be a useful tool for nephrologists in detecting CKD.

The limitation of the proposed model was that it had been tested on small data sets. To improve the model performance, significant volumes of increasingly sophisticated and representative CKD data will be collected in the future to detect disease severity. The clinical data to be collected from pathologist’s experts. The performance of the proposed model will be evaluated on a large clinical data set based on acid-base parameters, hyperparathyroidism, inorganic phosphorus concentration, and night urination in the future. Additionally, new features will be applied to get a broader perspective on the informative parameters related to CKD disease to test the prediction accuracy.

## Figures and Tables

**Table 2:** Characteristics of the UCI CKD data.

Features	Specification	Value
AGE	AGE (IN YEARS)	0–90
AL	ALBUMIN	0–5
ANE	ANAEMIA	NO, YES
APPET	APPETITE	POOR, GOOD
BA	BACTERIA	PRESENT, NOTPRESENT
BGR	BLOOD GLUCOSE RANDOM	0–490
BP	BLOOD PRESSURE	0–180
BU	BLOOD UREA	0–391
CAD	CORONARY ARTERY DISEASE	NO, YES
CLASS	CLASS	NOTCKD, CKD
DM	DIABETES MELLITUS	NO, YES
HEMO	HAEMOGLOBIN	0–17.8
HTN	HYPERTENSION	NO, YES
PC	PUS CELL	NORMAL, ABNORMAL
PCC	PUS CELL CLUMPS	PRESENT, NOTPRESENT
PCV	PACKED CELL VOLUME	0–54
PE	PEDAL EDEMA	NO, YES
POT	POTASSIUM	0–47
RBC	RED BLOOD CELLS	NORMAL, ABNORMAL
RC	RED BLOOD CELL COUNT	0–8
SC	SERUM CREATININE	0–76
SG	SPECIFIC GRAVITY	0–1.025
SOD	SODIUM	0–163
SU	SUGAR	0–5
WC	WHITE BLOOD CELL COUNT	0–26,400

**Table 3:** Experimental setup details.

Resource	Specification
Processor	Intel Core i5 Gen7
Random access memory	16 GB
Graphics processing unit	4 GB
Language	Python

**Table 4:** Hyper-parameter settings.

Hyper-Parameter	Setting
Epochs	850
Batch size	15
Dropout rate	0.5 to 0.1
Activation Function	relu
Activation output layer	sigmoid
Optimizer	Adam
Loss	binary_crossentropy

**Table 5:** Comparative analysis of the proposed model with existing classification techniques on CKD data set.

Method	Accuracy	Recall	Precision	F-Measure
Logistic Regression	0.99	1.0	0.98	0.99
*K*-Nearest Neighbor	0.92	0.88	0.98	0.92
Naïve bayes	0.95	0.92	1.00	0.95
Support Vector Machines	0.92	0.87	0.96	0.92
Decision Tree	0.97	0.95	1.00	0.97
Proposed Model	1.00	1.00	1.00	1.00

**Table 6:** Comparative analysis of the proposed model with existing models from the literature on the UCI data set.

Authors	Model	Accuracy (%)
Elhoseny et al. [19]	Ant Colony-based Optimization Classifier	95
Vasquez-Morales et al. [27]	Neural network	95
M Senan et al. [37]	*K*NN	98.33
Krishnamurthy et al. [38]	Convolutional Neural Networks	95.4
Polat, H et al. [45]	Support Vector Machine	98.5
Sarah A. et al. [46]	SAE and Softmax Regression	98
Proposed Model	Deep Neural Network	100

**Table 7:** The most critical risk factors from CKD data.

Risk Factor Name
Hemoglobin
Serum Creatinine
Red Blood Cell Count
Packed Cell Volume
Albumin
Specific Gravity
Hypertension

## Data Availability

Data were collected from UCI Machine Repository, CA, USA (http://archive.ics.uci.edu/ml (accessed on 18 June 2021). Source code and data are available at https://github.com/vsingh-fet/Deep_Neural (accessed on 28 December 2021).
